# TDP-43 loss-of-function triggers mitochondrial dysfunction and metabolic imbalance

**DOI:** 10.4103/NRR.NRR-D-25-00167

**Published:** 2025-07-05

**Authors:** Miriam Ceron-Codorniu, Anna Fernàndez-Bernal, Reinald Pamplona, Manuel Portero-Otín

**Affiliations:** Metabolic Pathophysiology Research Group, Universitat de Lleida-IRBLleida, Lleida, Spain

Neurodegenerative diseases are chronic, age-related disorders characterized by a relentless, irreversible, and selective loss of neurons in motor, sensory, or cognitive systems (Gao et al., 2019). Despite their heterogeneity, a common pathological feature across many of these diseases is the accumulation of aggregate-prone proteins. Particularly, the cytoplasmic aggregation in neurons of the Transactive response DNA-binding protein 43 (TDP-43), referred to as “TDP-43 proteinopathy,” is observed in most cases of amyotrophic lateral sclerosis (ALS) and frontotemporal dementia (Vanden Broeck et al., 2014). These inclusions are accompanied by the loss of TDP-43 nuclear localization, which leads to a loss of its main function regulating RNA metabolism. Besides, cytoplasmic inclusions have also been described to further contribute to this dysfunction by toxic gain of function mechanisms. Pathologically, both loss of function (LoF) and toxic gain of function disrupt many cellular processes including energy metabolism, mitochondria and endoplasmic reticulum function, autophagy, and protein-clearance systems (Gao et al., 2019). We have recently shown the effects of TDP-43 LoF on cellular metabolism and viability using human-induced pluripotent stem cell-derived motor neurons (hIPSC-MNs) and HeLa cells (Ceron-Codorniu et al., 2024). *TARDBP* loss decreased metabolic activity, impaired adenosine triphosphate (ATP) production, and enhanced superoxide production, suggesting that TDP-43 plays a crucial role in maintaining cellular bioenergetics. Furthermore, *TARDBP* depletion triggered a metabolic shift leading to lipid droplet accumulation and increased Long-chain-fatty-acid—CoA ligase 4 expression, while enhancing ferroptosis resistance.

**Figure 1:**
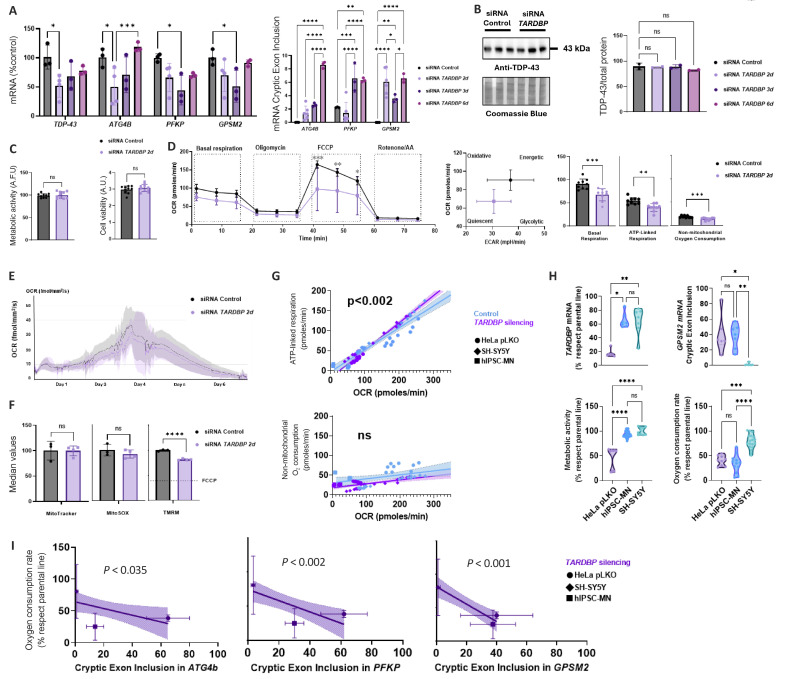
Cell type-specific metabolic effects of TARDBP loss. (A) Expression levels of *TARDBP*, *ATG4B*, *GPSM2*, and *PFKP* mRNA in SH-SY5Y cells 2, 3 and 6 days post-transfection with either control siRNA or *TARDBP*-targeting siRNA. The percentage of cryptic exon inclusion is also presented for *ATG4B*, *GPSM2*, and *PFKP*. (B) Protein levels of TDP-43 in SH-SY5Y cells 2, 3 and 6 days after siRNA-mediated *TARDBP* knockdown. (C) Assessment of metabolic activity (left) and cell viability (right) in SH-SY5Y cells 2 days post-transfection with either control or *TARDBP* siRNA. (D) OCR measurements, energy mapping, and comparisons of basal respiration, ATP-linked respiration, and non-mitochondrial oxygen consumption, performed using the Seahorse^TM^ analyzer and normalized to cell number by crystal violet assay, in SH-SY5Y cells 2 days after *TARDBP* knockdown. The experimental protocol for cellular respiration phenotype included three measurements per phase: (1) Basal respiration; (2) Oligomycin (1.5 μM); (3) FCCP (1.0 μM); (4) Rotenone/Antimycin A (0.5 μM each). (E) Oxygen consumption rate (OCR) analysis of SH-SY5Y cells transfected with control or *TARDBP* siRNA for 6 days. (F) MitoTracker^TM^, MitoSOX^TM^, and Image-iT^TM^ TMRM intensity after flow cytometry analyses in SH-SY5Y cells 2 days post-transfection with either control or *TARDBP* siRNA. FCCP (1.0 μM) was used as a positive control for mitochondrial uncoupling. (G) OCR exhibits a linear relationship with ATP production-linked oxygen consumption (upper panel) and non-mitochondrial oxygen consumption (lower panel). (H) The upper left panel illustrates experimental *TARDBP* depletion, which results in an increase in cryptic exon inclusion in selected mRNAs, such as *GPSM2* (upper right panel). The lower left panel highlights metabolic activity alterations associated with *TARDBP* expression, while the lower right panel depicts the concurrent decrease in OCR alongside diminished TDP-43 function. (I) Linear correlations between cryptic exon inclusion in specific mRNAs and changes in basal OCR levels. In A, differences were analyzed via two-way ANOVA with Sidak’s correction for multiple comparisons (*n* = 3 independent replicates per group; *P* = 0.23 for gene-silencing interaction, accounting for 13.9% of total variance; *P* = 0.35 for gene effects, 3.7% of total variance; *P* < 0.0001 for *TARDBP* depletion, 42.9% of total variance; effect sizes: *η*² = 0.13, 0.037 and 0.42, respectively). Cryptic exon accumulation analysis (*n* = 3 independent replicates per group) revealed *P* < 0.0001 for the gene-silencing interaction (29.6% of total variance); *P* = 0.08 for gene effects (2.06% of total variance); and *P* < 0.0001 for *TARDBP* loss (52.6% of total variance; *η*² = 0.3, 0.02, and 0.54, respectively). In B, differences were performed using one-way ANOVA with Dunnett’s correction for multiple comparisons (*n* = 2 independent replicates per group, *P* = 0.38; *η*² = 0.5). In C, comparisons were performed using Student’s *t*-test (*n* = 10 independent replicates per group, *P* = 0.61 for metabolic activity and *P* = 0.37 for cell viability, Cohen’s d = −0.19 and −0.49, respectively). In D, OCR differences were assessed using two-way repeated measures ANOVA with Sidak’s correction for multiple comparisons (*n* = 3 independent conditions per group; *P* = 0.024 for OCR-modulating drug interaction with *TARDBP* silencing, 4.5% of total variance; *P* < 0.0001 for OCR-modulating drug effects, 75% of total variance; *P* = 0.14 for TARDBP silencing effects, 5.6% of total variance; η² = 0.045, 0.75, and 0.05, respectively). Other OCR differences were examined using Student’s *t*-test (*n* = 9 independent conditions per group, Cohen’s d = 1.89, 1.86, and 1.93 for basal OCR, ATP-linked OCR, and non-mitochondrial OCR, respectively). In E, shaded areas represent 95% confidence intervals for OCR profiles. In F, differences were analyzed via Student’s *t*-test (*n* = 4 independent measurements of 10,000 cells per group, *P* = 0.34 for MitoSOX, *P* = 0.98 for MitoTracker and *P* < 0.0001 for TMRM, Cohen’s d −0.8, −0.019 and −11.09 respectively). In G and I, the relationship between OCR and ATP-linked respiration was evaluated via simple linear regression. In H, statistical differences were determined using the Kruskal-Wallis test with Dunn’s correction for multiple comparisons (*n* = at least 6–7 independent replicates per cell type; *η*² = 0.74, 0.64, 0.59, and 0.43 for *TARDBP* expression, metabolic activity, *GPSM2* cryptic exon inclusion, and OCR, respectively). In G, *P*-values correspond to differences in regression slopes induced by TARDBP silencing, while in I, *P*-values represent correlations between cryptic exon inclusion and OCR deficits resulting from *TARDBP* loss. Shaded areas denote 95% confidence intervals for regression lines. Equations of regression lines: (G) Left panel: Control condition, Y = 0.5489X – 2.301; *TARDBP* silencing, Y = 0.6843X – 17.08. (G) Right panel: Control condition, Y = 0.1007X + 30.66; *TARDBP* silencing, Y = 0.09951X + 18.14. (I): *ATG4B*, Y = −0.5255X + 64.20; *PFKP*, Y = −0.8167X + 74.32; *GPSM2*, Y = −1.303X + 81.47. Western blot analyses were normalized to total protein content, quantified via densitometric analysis of Coomassie Blue-stained protein loads. Normality of data distributions was assessed using the Shapiro-Wilk or Kolmogorov-Smirnov tests. Significance levels are denoted as follows: ns: not significant; *P < 0.05, **P < 0.01, ***P < 0.001, ****P < 0.0001. Metabolic activity and cell viability measurements were obtained via PrestoBlue^TM^ and crystal violet assays as previously described (Ceron-Codorniu et al., 2024). ATP concentrations were inferred from OCR data, following the manufacturer’s guidelines. mRNA quantifications were normalized to *GAPDH* mRNA levels, except for cryptic exon analyses in *GPSM2*, *ATG4B*, and *PFKP*, where cryptic exon inclusion was determined as the ratio of cryptic exon-containing mRNA to canonical mRNA, as previously described (Ceron-Codorniu et al., 2024; Torres et al., 2024). Control siRNA sequence: sense 5′-UAA GGC UAU GAA GAG AUA-3′; antisense 5′-GUA UCU CUU CAU AGC CUU-3′; *TARDBP* siRNA sequence: sense 5′-GCA AAG CCA AGA UGA GCC-3′; antisense: 5′-AGG CUC AUC UUG GCU UUG-3′. Unpublished data. AA: Antimycin A; A.F.U: arbitrary fluorescence units; ANOVA: analysis of variance; ATP: adenosine triphosphate; A.U.: arbitrary units; ECAR: extracellular acidification rate; FCCP: carbonylcyanide-p-trifluoromethoxyphenylhydrazone; hIPSC-MN: human induced pluripotent stem cell – motoneuron; mRNA: messenger RNA; siRNA: small interference RNA.

To further investigate the pathophysiological mechanisms underlying TDP-43 dysfunction, we present the results of silencing TDP-43 in SH-SY5Y neuronal cells in this update. We compared multiple cellular models of TDP-43 LoF including hIPSC-MNs, HeLa cells, and SH-SY5Y cells. To minimize artifacts from gene silencing methods, *TARDBP* expression was reduced in each model using different approaches: lentiviral infection, doxycycline-inducible silencing, and small interference RNA (siRNA) transfection, achieving silencing efficiencies of 50%, 90%, and 40%, respectively. In the SH-SY5Y model, *TARDBP* silencing resulted in increased inclusion of cryptic exons in *ATG4B*, *GPSM2*, and *PFKP* (phosphofructokinase, platelet) mRNA – hallmarks of TDP-43 dysfunction (**Figure 1A**) – without significant changes in TDP-43 protein levels (**Figure 1B**). Global cellular health, assessed by metabolic activity and cell viability, showed no significant changes (**Figure 1C**). However, the oxygen consumption rate (OCR) was significantly reduced, affecting basal respiration, ATP-linked respiration, and non-mitochondrial respiration (**Figure 1D** and **E**). Additionally, the mitochondrial spare capacity, essential for responding to high energy demands or stress conditions, was particularly impaired upon treatment with the oxidative phosphorylation uncoupler carbonylcyanide-p-trifluoromethoxyphenylhydrazone (FCCP). Despite that the common normalization procedure for Seahorse-produced data is adjustment for cell number and surrogate measurements, as a limitation we could not exclude changes in mitochondrial mass or mitochondrial activities as contributors to OCR changes. However, TDP-43 loss did not cause changes in mitochondrial mass or reactive oxygen species (ROS) production, as evidenced by Mitotracker^TM^ and MitoSOX^TM^ (**Figure 1F**). Furthermore, no changes were observed in voltage-dependent anion-selective channel (VDAC) levels following TDP-43 knock-down (data not shown), suggesting that the changes in OCR were not fully explained by decreased mitochondrial number or mass. Nonetheless, mitochondrial membrane potential was significantly reduced, as indicated by *Image-iT*^TM^ TMRM (**Figure 1F**), supporting alteration in respiratory complexes explaining decreased OCR. Of note, in other contexts, TDP-43 is required for correct respiratory complex assembly (Wang et al., 2016).

Indeed, the relationship between OCR and ATP production, a surrogate measure of mitochondrial coupling efficiency, was found to be dependent on *TARDBP* expression (**Figure 1G**). Cells with silenced *TARDBP* required steeper OCR-ATP slopes, indicating enhanced mitochondrial efficiency, lower uncoupling protein activity, or increased availability of substrates such as NADH and FADH_2_. Notably, these effects were primarily mitochondrial, as non-mitochondrial OCR remained unchanged across all models. When comparing *TARDBP* expression levels across the three models – HeLa, hIPSC-MNs, and SH-SY5Y – silencing was most pronounced in HeLa cells, followed by hIPSC-MNs and SH-SY5Y cells (**Figure 1H**). However, cryptic exon inclusion in *GPSM2* mRNA was similarly high in HeLa and hIPSC-MN models but markedly lower in SH-SY5Y cells, suggesting that the degree of TDP-43 dysfunction does not directly correlate with *TARDBP* expression levels. Instead, it appears to depend on cell-type-specific sensitivity, with hIPSC-MNs exhibiting the highest sensitivity to TDP-43 LoF. In terms of metabolic activity, only the HeLa model showed a significant reduction following TDP-43 silencing, highlighting the limited sensitivity of this parameter to TDP-43 dysfunction. In contrast, OCR measurements closely correlated with TDP-43 dysfunction levels, showing similar impairments in the HeLa and hIPSC-MN models, but minimal effects in SH-SY5Y cells. These observations suggest that cellular respiration is intimately linked to TDP-43 dysfunction, as supported by the negative correlation between cryptic exon inclusion and OCR (**Figure 1I**). Overall, we interpret that the effects are both cell and silencing dose independent: i.e., the loss of TDP-43 would not only generate decreased viability potential, but also decreased proliferation capacity (which in cell lines would explain part of the results of OCR), decreased ATP production, and increased oxidative stress, to name a few. These changes would impact different cells, being differentiated cells such as motor neurons quite sensitive to the diminished ATP production, and cell lines being more resilient (such as SHSY-5Y or HeLa cells). Thus, these later would require stronger *TARDBP* silencing to generate similar metabolic consequences.

As previously described, TDP-43 LoF adversely affects both neuronal and non-neuronal cells (e.g., glia and muscle), as well as the viability, development, and lifespan of animal models, including worms, flies, fish, and rodents (Vanden Broeck et al., 2014). In this and prior studies (Ceron-Codorniu et al., 2024), we demonstrate that viability, respiration, and lipid metabolism are closely linked to TDP-43 function, with a particular focus on its impact on mitochondrial dysfunction and oxygen consumption. Notably, the degree of TDP-43 dysfunction is influenced not only by residual TDP-43 levels but also by cell type, with hIPSC-MN exhibiting more pronounced effects. This heightened vulnerability of the hIPSC-MN model may stem from their primary culture nature (in contrast to immortalized cell lines) or an increased sensitivity to TDP-43 dysfunction, given the high energy demands of neuronal cells.

Mitochondrial dysfunction is a hallmark of ALS pathophysiology and has been extensively associated with TDP-43 in the literature (Gao et al., 2019). TDP-43 has been found to localize to the inner mitochondrial membrane and regulate nuclear-encoded mitochondrial genes (Wang et al., 2016). Specifically, TDP-43 LoF impairs mitochondrial morphology, axonal and dendritic trafficking, mitochondrial membrane potential, and increases ROS production. It also disrupts mitochondrial functions such as oxidative phosphorylation and ATP production (Gao et al., 2019). Mechanistically, TDP-43 binds to mRNAs codifying peptides within mitochondrial complexes, including ND3, ND5, ND6, COXI, COXIII, CYTB, and A6 (Wang et al., 2016). Among these, ND3 and ND6 are of particular interest as TDP-43 represses their transcription and its absence results in their upregulation (Wang et al., 2016). These subunits are encoded by mitochondrial DNA and form part of the core transmembrane region of Complex I. Overexpression of ND6 may disrupt Complex I assembly, as it is required for recruiting ND1 and ND5-containing intermediates. Similarly, ND3 dysregulation has been implicated in processes associated with ALS pathophysiology, including enhanced sensitivity to apoptosis, oxidative damage, fatty acid metabolism, hypoxia, and mitophagy (McFarland et al., 2004).

Notably, TDP-43 has been shown to interact with several key mitophagy elements, including prohibitin 2, voltage-dependent anion-selective channel 1 (VDAC-1) and parkin (PRKN) (Ravanidis and Doxakis, 2020). TDP-43 LoF could impair mitophagy through different mechanisms: (1) reducing the interaction with VDAC1 on the outer mitochondrial membrane, potentially disrupting PRKN-mediated ubiquitination and hindering the clearance of damaged mitochondria; (2) decreasing prohibitin 2 levels, leading to reduced interaction with Microtubule-associated protein 1A/1B-light chain 3 and accumulation of damaged mitochondria; and (3) increasing PRKN expression, which may override proteasomal activity and disrupt mitophagy regulation (Ravanidis and Doxakis, 2020). Additionally, TDP-43 LoF disrupts mitochondrial dynamics by altering fusion and fission proteins, including mitofusin 1, mitofusin 2, mitochondrial fission 1 protein, mitochondrial dynamin like GTPase OPA1 and dynamin-related protein 1. This imbalance may lead to elongated mitochondria and potential dysfunction, further contributing to impaired mitophagy (Ravanidis and Doxakis, 2020). This aligns with the mitochondrial enlargement observed in the HeLa LoF model in our previous study (Ceron-Codorniu et al., 2024), although no significant differences in OPA1 and mitochondrial fission 1 protein expression levels were found.

Of note, cryptic exons in selected mRNA, such as *ATG4B*, may impair the function of derived proteins. For example, reduced *ATG4B* levels could disrupt macroautophagy, particularly autophagosome membrane formation, further compromising mitochondrial quality control. This may explain the presence of aberrant vesicular-like structures surrounding damaged mitochondria observed in our previous work (Ceron-Codorniu et al., 2024). Additionally, TDP-43 dysfunction also affects glucose metabolism due to decreased levels of PFKP, a key glycolytic enzyme. PFKP catalyzes the conversion of fructose-6-phosphate to fructose-1,6-bisphosphate, a critical regulatory step in glycolysis. Loss of PFKP reduces glycolytic flux, ATP production, and the balance of metabolic intermediates, such as glucose-6-phosphate, fructose-6-phosphate, glyceraldehyde-3-phosphate, and pyruvate, which are essential for maintaining cellular energy homeostasis. Moreover, recent data suggest that mitochondrial dysfunction in ALS extends beyond TDP-43 pathology, as seen in superoxide dismutase 1 (*SOD1*) mutations, which alter Complex I function and electron flux, leading to increased free radical production and metabolic switch towards free fatty acids (Larrea et al., 2025). Whether similar mechanisms apply to TDP-43-related mitochondrial dysfunction remains to be explored.

Consistent with the metabolic consequences of mitochondrial dysfunction, we observed increased neutral lipid staining, we evidenced increased neutral lipid staining in HeLa LoF cells, which may be compatible with enhanced lipid droplet (LD) accumulation (Ceron-Codorniu et al., 2024). In ALS, LD accrual is closely linked to mitochondrial dysfunction and impaired autophagy, creating a cycle of metabolic stress that exacerbates motor neuron degeneration (Dodge et al., 2020). Mitochondria rely on balanced lipid metabolism to maintain membrane integrity and energy production. However, excessive storage of cholesterol esters and triacylglycerols in LD may disrupt lipid homeostasis, leading to mitochondrial membrane instability, reduced oxidative capacity, and increased ROS production. This dysfunction further impairs mitochondrial energy metabolism regulation, increasing neuronal vulnerability to stress. At the same time, disrupted autophagy–a crucial mechanism for cellular quality control–hinders the proper degradation of damaged mitochondria and accumulated LDs. Consequently, toxic lipid byproducts such as lysophosphatidylcholine accumulate, further damaging cellular membranes, triggering inflammatory responses, and amplifying cellular stress, ultimately driving neurodegeneration in ALS.

Overall, neurodegenerative diseases related to TDP-43 proteinopathy, such as ALS and frontotemporal dementia, involve cytoplasmic aggregation and nuclear clearance. To investigate this, we generated TDP-43 LoF cellular models (hIPSC-MNs, HeLa, and SH-SY5Y cells) that exhibit disrupted RNA metabolism and mitochondrial function, leading to energy deficits, impaired autophagy, and endoplasmic reticulum stress (Ilieva et al., 2007; Ceron-Codorniu et al., 2024; Torres et al., 2024). Despite some limitations due to not including rescue experiments as those described before (Ceron-Codorniu et al., 2024), using these models, we demonstrated that TDP-43 LoF reduces mitochondrial respiration and spare respiratory capacity, with cell-type-dependent effects on cryptic exon inclusion and metabolic activity. In common with other ALS contexts, TDP-43 regulates mitochondrial gene expression, and its loss disrupts Complex I assembly, increases ROS production, and impairs glucose metabolism. Overall, these findings highlight the central role of mitochondrial dysfunction in TDP-43 pathology and its contribution to neurodegeneration. We believe that the data presented here may help elucidate the potential interaction between toxic gain- and loss-of-function mechanisms in TDP-43 proteinopathy, both of which may contribute to pathogenicity.

*This work was funded by the “Instituto de Salud Carlos III” (PI 17–000134, PI 20–0155, PI23/00176) and the “Diputació de Lleida” (PP10601—PIRS2021) to MPO; Also from the “Diputació de Lleida” (PP10605—PIRS2021) and the “Generalitat de Catalunya”: Agency for Management of University and Research Grants (2021SGR00990) to RP; Support was also received in the form of a FUNDELA Grant, “RedELA-Plataforma Investigación” and the “Fundació Miquel Valls” (Jack Van den Hoek donation) (to MPO); MCC is a “University of Lleida” PhD fellow. AFB is a “Fundació LaCaixa” postdoctoral fellow. These funding bodies had no roles in the design of the study, collection, analysis, interpretation of data, and in writing the manuscript.*
